# The Nordic maintenance care program: maintenance care reduces the number of days with pain in acute episodes and increases the length of pain free periods for dysfunctional patients with recurrent and persistent low back pain - a secondary analysis of a pragmatic randomized controlled trial

**DOI:** 10.1186/s12998-020-00309-6

**Published:** 2020-04-21

**Authors:** Andreas Eklund, Jan Hagberg, Irene Jensen, Charlotte Leboeuf-Yde, Alice Kongsted, Peter Lövgren, Mattias Jonsson, Jakob Petersen-Klingberg, Christian Calvert, Iben Axén

**Affiliations:** 1 Karolinska Institutet, Institute of Environmental Medicine, Unit of Intervention and Implementation Research for Worker Health, Stockholm, Sweden; 2 Institute for Regional Health Research, University of Southern Denmark, Odense, Denmark; 3 Nordic Institute of Chiropractic and Clinical Biomechanics, Odense, Denmark; 4 Department of Sports Science and Clinical Biomechanics, University of Southern Denmark, Odense, Denmark; 5 Private practice, Stockholm, Sweden; 6 Private practice, Lidköping, Sweden; 7 Private practice, Borlänge, Sweden; 8 Private practice, Falkenberg, Sweden

**Keywords:** Low back pain, Timing, Dose, Chiropractic, Prevention, Maintenance care, Manual treatment, Effect, Secondary prevention, Tertiary prevention

## Abstract

**Background:**

A recent study showed that chiropractic patients had fewer days with bothersome (activity-limiting) low back pain (LBP) when receiving care at regular pre-planned intervals regardless of symptoms (‘maintenance care’, MC) compared to receiving treatment only with a new episode of LBP. Benefit varied across psychological subgroups. The aims of this study were to investigate 1) pain trajectories around treatments, 2) recurrence of new episodes of LBP, and 3) length of consecutive pain-free periods and total number of pain-free weeks, for all study participants as well as for each psychological subgroup.

**Methods:**

A secondary analysis of data from a randomized controlled trial of patients (*n* = 319) seeking chiropractic care for recurrent or persistent LBP used 52 weekly estimates of days with bothersome (activity-limiting) LBP. First, a generalized estimating equations analysis was used to compare the pain trajectory before and after the initial treatment in every new treatment period. Thereafter, a time-to-event analysis (using Cox regression) estimated time to/risk of a new LBP episode. The analyses were performed on i) all study participants and ii) separately for each psychological sub-group (named adaptive copers, interpersonally distressed and dysfunctional) classified by the West Haven-Yale Multidimensional Pain Inventory.

**Results:**

Patients receiving MC had flat pain trajectories around each new treatment period and reported fewer days with pain compared to patients receiving the control intervention. The entire effect was attributed to the dysfunctional subgroup who reported fewer days with activity limiting pain within each new LBP episode as well as longer total pain-free periods between episodes with a difference of 9.8 weeks (CI 95% 3.3, 16.3) compared to the control group. There were no differences in the time to/risk of a new episode of LBP in either of the subgroups.

**Conclusion:**

Data support the use of MC in a stratified care model targeting dysfunctional patients for MC. For a carefully selected group of patients with recurrent and persistent LBP the clinical course becomes more stable and the number of pain-free weeks between episodes increases when receiving MC. Understanding how subgroups of patients are likely to be affected by MC may help align patients’ and clinicians’ expectations based on realistic outcomes.

**Trial registration:**

Clinical trials.gov; NCT01539863; February 22, 2012.

## Background

Low back pain (LBP) is a recurrent and persistent non-communicable condition ranked highest in in the world in terms of disability and resulting in a major societal burden [1, 2]. Secondary or tertiary preventive strategies are needed to manage a global health challenge such as LBP. A modest number of interventions have been shown to be effective in managing and treating recurrent and persistent LBP [3]. However, only exercise and exercise combined with education have been shown to reduce the number of new episodes [4–7].

Manual treatments performed by chiropractors have been found to be effective in reducing pain intensity and disability for patients with LBP and are recommended in current practice guidelines [8, 9]. About 98% of all chiropractors who are members of the Swedish Chiropractic Association consider a treatment strategy known as maintenance care (MC) to be clinically useful and beneficial for patients with recurrent and persistent musculoskeletal pain [10]. MC is a secondary/tertiary treatment strategy where patients are treated at regular intervals over a lengthy period of time with the aim of preventing future episodes (secondary prevention) or managing persistent pain (tertiary prevention) [11, 12]. In a joint initiative, researchers in Sweden, Denmark and Finland have investigated the frequency, indications and content of MC [13–21]. Based on their findings a multicenter pragmatic randomized clinical trial was conducted in Sweden from 2012 to 2016 [22, 23]. The trial found that MC was effective in reducing the total number of days with activity limiting (bothersome)LBP during a 12 month period compared to treatment ‘when needed’ [23]. In total the MC group (*n* = 163) reported 12.8 (95% CI: 10.1, 15.5) fewer days with activity limiting LBP compared to the control group (*n* = 158) and received 1.7 (95% CI: 1.8, 2.1) more treatments.

In a secondary analysis of the data from the RCT it was found that psychological sub-groups defined by the Swedish version of the West Haven-Yale Multidimensional Pain Inventory (MPI-S) could identify responders and non-responders to MC [24].

The MPI-S is a comprehensive patient-reported screening instrument based on the cognitive behavioral model which was developed to capture and measure the experience of chronic pain [25]. The Swedish version of the instrument has 34 items that can be used to classify patients into three clinically relevant and valid subgroups: adaptive copers, interpersonally distressed and dysfunctional [26–29]. Adaptive copers (AC) are characterized by low pain severity, low interference with everyday life, low life distress, a high activity level and a high perception of life control. Interpersonally distressed (ID) tend to perceive negative responses by spouses or significant others to their pain behavior and complaints, for example not being supportive/helpful, and expressing irritation, frustration and anger. Dysfunctional (DYS) individuals are characterized by high pain severity, marked interference with everyday life, high affective distress, low perception of life control and low activity levels.

Patients who were classified as dysfunctional reported statistically and clinically significantly fewer days with bothersome (activity-limiting) LBP (− 30.0; 95% CI: − 36.6, − 23.4) in the MC group compared to the control group. On the other hand, patients who were classified as adaptive coper reported a worse outcome where the number of days with bothersome (activity-limiting) LBP was higher (10.7; 95% CI 4.0, 17.5) in the MC groups even though they received a greater number of treatments (3.9; 95% CI: 3.5, 4.2) compared to the control group. There is now compelling evidence that stress the importance of careful the selection of patients for MC to include those who have recurrent or persistent low back pain, a dysfunctional psychological profile and a good initial response to manual therapy.

It is imperative that we undertake research and implement preventive strategies with clear and precise definitions of target populations and disease states. The definition of new episodes for a recurrent disorder such as LBP requires a clear definition of ‘recovery’. De Vet et al. suggested that an episode of LBP should be defined as at least 24 h of pain with at least four pain-free weeks before and after [30]. The prevalence of four consecutive pain-free weeks has been studied in a number of populations and found to represent a good marker of non-episodes [31–33]. In addition, there is a logical inverse dose-response relationship between consecutive numbers of pain-free weeks and previous duration of pain [31]. Thus, a period of four pain-free weeks can be used as a marker of recovery, necessary when defining an episodes [30, 31].

The optimal dose of treatments for the long-term management of recurrent and persistent LBP has not been established. In fact, we are not sure whether the effect of MC is a matter of dose (a higher number of visits yielding a reduction of pain irrespective of when the visits occur) or timing (preventing the number of episodes or reducing the number of days within each episode by timing the treatment before or in the early stage of the new event). If the effect of MC is related to the timing of visits, patients who consult at different stages in their long-term pain trajectory and pain around the treatments would have different outcome profiles (i.e. MC treatments should take place before the recurrence of pain and control treatments after the recurrence of pain). The previous secondary analysis of these data, which considered outcome in relation to psychological sub-groups [24], suggested that the outcome is not dependent on number of treatments because the effect of MC seen in the dysfunctional subgroup was achieved with the same number of visits as in the control group. Indeed, a larger number of MC visits could potentially be harmful for some patients, given the outcome for the adaptive coper subgroup. To further our understanding of how MC affects LBP, another secondary analysis of the data from the RCT was performed to investigate the trajectory of pain episodes, the occurrence of (time to/risk of) new episodes of LBP and pain-free periods in relation to patients receiving care when experiencing a symptomatic relapse.

## Method

### Aim

The overall aim of this project was to explore how MC affects the bothersome (activity-limiting) LBP around treatment periods, new episodes of LBP and pain-free periods between episodes as compared to patients receiving care when experiencing a symptomatic relapse. The specific objectives were to compare the following for i) all participants in the trial as well as the ii) psychological subgroups defined by the MPI-S instrument:

1. The pain trajectory before and after a single visit or the first visit in every new treatment period.
2. The time to and risk of a new episode following the first recovery period.
3. The length of consecutive pain-free periods and total number of pain-free weeks during the study period.

### Design

This study is a secondary analysis of data from a pragmatic, multicenter, investigator- and assessor- blinded randomized controlled trial with a two-arm parallel design [22–24]. The trial was based on the findings of the Nordic Maintenance Care program and made use of all the current evidence in the field [13–21]. The primary aim of the trial was to measure the differences in outcome of MC on patients with recurrent or persistent LBP. A total of 35 licensed chiropractors from the Swedish Chiropractic Association requited patients in a nationwide practice-based research-network. The follow-up period was 52 weeks and the primary outcome of the trial was “number of days with bothersome (activity-limiting) LBP” measured using weekly text-messages, SMS. The trial design has been extensively reported in a published protocol paper [22] and in two previous papers evaluating the outcomes of MC [23, 24].

### Participants

Patients with recurrent and persistent LBP, who had responded favorably to an initial course of chiropractic care, were recruited in a consecutive sequence between 2012 and 2016 from chiropractic clinics that were part of a nationwide practice-based research network in Sweden. Patients were screened in a three-stage procedure, i.e. at the first visit (baseline 1), the fourth visit (baseline 2), and at study start (baseline 3). See Table 1 for the inclusion criteria at each stage of the trial. Both clinicians and patients received follow-up questionnaires at 12 months. During the inclusion procedure (baseline 1, 2, and 3) patients followed a normal treatment pathway and were included in the trial (i.e. randomly allocated to one of the treatment arms), when the clinician would, as in clinical practice, either schedule the patient for MC or end the current treatment plan.

**Table 1:** Eligibility Screening in a study of patients with recurrent or persistent low back pain from a randomized clinical trial investigating the comparative effectiveness of chiropractic maintenance care

Time point	Inclusion criteria	Exclusion criteria
Baseline 1 (1st visit)	Age 18–65 years. LBP with or without leg pain for altogether more than 30 days during the previous year. Previous episodes. Access to a mobile phone. Ability to send and receive SMS (text messages).	Pregnancy. Chiropractic treatment less than 3 months previously. Completely subsidized treatment from 3rd party payer. Serious pathology (i.e. acute trauma, cancer, infection, cauda equina, osteoporosis, vertebral fractures) or other contraindications to manual therapy.
Baseline 2 (4th visit)	Self-rated “definitely improved”	
Baseline 3 (Study start)	Interval between treatments (after the 4th visit) can be scheduled 1 month or more.	

*LBP* non-specific low back pain (Table reproduced from study protocol, approved by authors [22])

One of the key components and inclusion criterion of the trial was to select patients who reported clinical benefit from chiropractic care during the initial treatment. Initial clinical benefit was assessed at the 4th visit using the global perceived improvement scale (single question with one answer in 5 levels: definitely worse, probably worse, unchanged, probably improved, definitely improved). If the patients stated that they were “definitely improved” by the fourth visit, initial clinical benefit was considered evident. Previous research has shown that using the global perceived improvement scale in this way can predict a favourable long-term outcome from chiropractic care at 3, 6, and 12 months [34–37]. According to research from the Nordic Maintenance Care program, early favorable treatment response is an indication for recommending MC and this step was therefore a core component of the design to reinforce the pragmatic nature of the trial [10, 16–18, 20, 21].

Once patients were screened at the 3 baseline steps and considered candidates for the study, they were invited to partake in the study and asked to sign an informed consent form with information about the trial. It was also made clear to patients in the written information that they were free to withdraw from the project at any point without detriment to their relationship with the clinician.

### Interventions

The two arms of the trial have been described as MC (preventive treatment, i.e. clinician-controlled) and control (symptom-guided treatment, i.e. patient controlled). *The MC group* received regularly scheduled treatments, 1–3 months apart during the 52 weeks of follow-up. If the patient had a relapse of pain, a more intense period of visits was scheduled until the patient once again was suitable for a MC plan. *The control group* was instructed to seek care only if they experienced a symptomatic relapse. In such cases a period of frequent visits would be scheduled until maximum benefit was reached, after which the patients were again instructed to seek care if the LBP reoccurred. Content of care within the two treatment arms were similar, consisting of spinal manipulation, information/advice and soft tissue treatment, as was the level of attention given to the patients [23]. Both groups had 50% of the treatment fee subsidized by the participating clinicians. Prior to the start of the trial, clinicians were given a carefully written study protocol with instructions about the trial procedures. Much effort, by means of physical meetings and telephone contact, was made to ensure that all clinicians had understood and would comply with the study procedures.

### Randomization and information to participants

A statistician at Karolinska Institutet generated 40 permuted blocks of 10 participants with a 1:1 allocation ratio according to a randomization schedule. Each clinician received 10 opaque, sealed envelopes containing information about the procedure the patient had been randomly allocated to. The envelopes were opened in front of the patient at the initial visit of the study. Clinicians were instructed to inform patients that 1) the two treatment arms were different procedures currently being used in practice, and 2) there was no evidence to suggest that one strategy was more effective than the other [22].

### Outcome variables

The primary outcome of the trial was number of days with bothersome (activity-limiting) LBP. It was recorded weekly using an automated text message system (SMS-track®) [38–40] that allowed the researchers to monitor the data collection process from a web-interface in real-time. Thus, patients received the message: *“On how many days during the past week were you bothered by your lower back (i.e. it affected your daily activities or routines)? Please answer with a number between 0 and 7”.* If the patient failed to respond to the weekly SMS, an automatic reminder was sent after 48 h. If the patient failed to respond after 2 weeks, a research assistant called the patient to answer any concerns that may have caused the patient not to respond.

At follow-up the chiropractor filled in a questionnaire where they were asked to review each patient’s clinical record and document the dates of all visits during the study period. This information was then used to model the trajectory analysis.

To apply the definition by de Vet et al. [30]. as closely as possible to our data, a *pain-free week* was classified as a week with ≤1 day with activity-limiting LBP in the time-to-event analysis. The classification of pain-free weeks as “≤2 days with bothersome (activity-limiting) LBP” instead of “<2 days with bothersome (activity-limiting) LBP” minimized the risk of misclassification of weeks with temporary short (< 24 h) exacerbations of LBP as episodes. Thus, a *new pain episode* was defined as a week with ≥2 [2–7] days of bothersome (activity-limiting) LBP preceded by ≥4 consecutive pain-free weeks according to the suggested classification above. A pain episode was considered ongoing until ≥4 consecutive pain-free weeks were recorded.

A *treatment-period* was defined as a single visit or as the first of a series of visits with an interval of ≤2 weeks apart, followed by ≥3 consecutive weeks without any visits to the chiropractor (1 month between visits). MC visits were scheduled 1–3 months apart according to the normal procedures used in clinical practice [41] as reported in the study protocol [22]. The definition of treatment period was chosen to include each MC visit as a new period, any interval between treatments of less than 1 month was considered an active treatment period [41]. In the analysis, each study participant contributed with all treatment periods during the 52-week study period.

A *pain-trajectory* was defined as “the number of days with bothersome (activity-limiting) LBP per week, during a 7-week period around the week of a single visit or the first visit in a new treatment period” (3 weeks before and 3 weeks after the treatment). The trajectory (mean number of days with pain) for each of the 7 weeks for all single visits or new treatments periods for per group was estimated.

### Psychological sub-groups

The MPI-S subgroups have been extensively described in the previous publication, which reported the sub-group evaluation of effect of MC [24]. Patients were classified by the MPI-S instrument, as part of the first visit screening procedure (baseline 1).

### Statistical methods

The data from the RCT was analyzed with an intention to treat analysis. Estimates in this study were reported with arithmetic means and 95% confidence intervals. Only individuals who had > 12 weeks of complete SMS data and follow up data for number and dates of visits were included in the analyses.

Data relating to the first objective were analyzed in a longitudinal model which looked at the pain trajectory around the initial visit of each treatment period. Mean number of days with bothersome (activity-limiting) LBP was calculated for each group and for each week during the 7-week treatment period (3 weeks before the visit, week of the visit and 3 weeks after). If two visits were separated by 3 weeks without visits (i.e. defined as two separate treatment periods in the statistical model), the analysis would then double count the last 3 weeks [5–7] in the first treatment period and the first 3 weeks [1–3] in the following treatment period. The mean number of visits for the whole cohort over 12 months was six visits, so the probability of double counting weeks without pain (as described above) was considered low, with little risk of distorting the results. Data were analyzed by means of a Generalized Estimating Equations (GEE) linear regression model, using an appropriate correlation structure (the best ‘quasi-likelihood under the independence model criterion (QIC)’ value for each strata) and a robust estimator of variance (robust to non-constant error variance). The analysis included treatment group and time (both as study period (52 weeks) and treatment period (7 weeks)) as continuous variables. These covariates were tested as single variables in combinations as well as interaction terms. Co-variates were excluded based on test of model effects, and all variables with a significance level of *p* < 0.20 were considered for the regression model. The best model structure was further decided based on goodness of fit values (QIC-value). All remaining variables in the final models, chosen based on the best goodness of fit, ended up with significance levels of *p* < 0.05. Data were also analyzed using a Mixed Model framework. The results of the two procedures were very similar; GEE was chosen rather than the mixed model framework because of its ease of use. Results were presented in graphs, with lines representing mean values and estimated group differences for each week of days with bothersome pain, illustrating pain trajectories for the control and intervention groups.

Data relating to the second objective of this study were analyzed in a time-to-first event model using Cox regression in a survival analysis, estimating the Hazard Ratio of experiencing new LBP episodes. Only individuals who initially reached a recovered state (≥4 consecutive weeks free from bothersome LBP), were included in the Cox regression.

In the third objective, differences between groups for i) time-to-event and ii) length of consecutive pain-free periods and total number of pain-free weeks were tested using ANOVA.

Data were analyzed for all participants (included in the primary analysis [23]), in line with the intention to treat analysis as well as stratified according to the 3 MPI-S subgroups (adaptive copers, interpersonally distressed and dysfunctional included in the subgroup analysis [24]).

## Results

As previously reported, 321 participants were randomly allocated into the two study groups after the initial inclusion procedure [23]. During the study period, 16,692 SMS messages were sent with a response rate of 98.9%. Two participants had ≥12 weeks of missing SMS data and were excluded from the dataset, leaving 319 individuals to be included in the analysis [23].

Two hundred and fifty-four participants had at least one treatment and could be included in the longitudinal pain trajectory analysis. A total of 1103 treatment-period sequences were identified during the 12-month study period (344 control, 759 MC) and were used for the analyses. In all, 250 participants reached at least one recovered state and could be included in the time-to-first-event analysis.

The trajectory analysis captured a sample with fewer females than the samples in the total and the survival analyses. There were also somewhat fewer individuals classified as adaptive copers in the control group for the trajectory analysis than for the total and the survival analyses. Other than these differences the samples across analysis groups and across treatment groups were similar. Descriptive data for the participants included in each analysis are reported in Table 2.

**Table 2:** Descriptive data of patients with recurrent or persistent low back pain from a randomized clinical trial investigating the outcomes of chiropractic maintenance care

Variable ^a^		Group	All n = 319 (MC, *n* = 161; Control, *n* = 158)	Survival analysis, *n* = 250 (MC, *n* = 127; Control, *n* = 123)	Trajectory analysis, *n* = 254 (MC, *n* = 145; Control, *n* = 109)
Age at study start, mean (SD)		MC	43.4 (11.2)	43.0 (11.3)	43.5 (11.3)
		Control	43.1 (13.2)	42.4 (13.3)	42.6 (13.6)
Female, % (n)		MC	63.5 (94)	64.7 (77)	37.9 (50)
		Control	60.3 (85)	57.5 (65)	37.0 (37)
Pain intensity, 0–10, mean (SD)	1st visit	MC	5.4 (2.1)	5.2 (2.1)	5.4 (2.0)
		Control	5.4 (2.1)	5.3 (2.1)	5.6 (2.1)
	4th visit	MC	2.4 (1.8)	2.3 (1.8)	2.6 (1.8)
		Control	2.3 (1.7)	2.2 (1.7)	2.3 (1.8)
	Study start	MC	2.1 (1.5)	1.8 (1.4)	2.1 (1.5)
		Control	2.2 (1.8)	2.0 (1.6)	2.2 (1.7)
EQ5D score baseline, mean (SD)		MC	0.68 (0.23)	0.67 (0.24)	0.69 (0.21)
		Control	0.70 (0.20)	0.71 (0.20)	0.71 (0.19)
MPI Subgroup, % (n)	AC	MC	29.8 (48)	31.0 (40)	32.0 (46)
		Control	28.3 (45)	32.5 (40)	23.9 (26)
	ID	MC	20.5 (33)	21.3 (27)	20.0 (29)
		Control	18.2 (29)	18.7 (23)	22.0 (24)
	DYS	MC	30.4 (49)	29.1 (37)	32.0 (46)
		Control	30.2 (48)	29.3 (36)	34.9 (38)
	Missing	MC	19.3 (31)	18.0 (23)	24 (17)
		Control	23.3 (37)	19.5 (24)	21 (19)
RMDQ Score (study start), mean (SD)		MC	4.9 (4.0)	4.4 (3.9)	4.9 (4.1)
		Control	4.7 (4.1)	4.3 (4.1)	4.9 (3.8)

^a^**,** No imputation has been made for missing data, mean values and percentages are based on true responses for each variable; *MC* Maintenance Care; *SD* Standard deviation; *n* number of participants; *MPI* West Haven-Yale Multidimensional Pain Inventory; *AC* Adaptive Coper; *ID* Interpersonally Distressed; *DYS* Dysfunctional; *RMDQ* Roland Morris Disability Questionnaire

### Pain trajectory analysis before and after a single visit or the first visit in every new treatment period

**Fig. 1:**
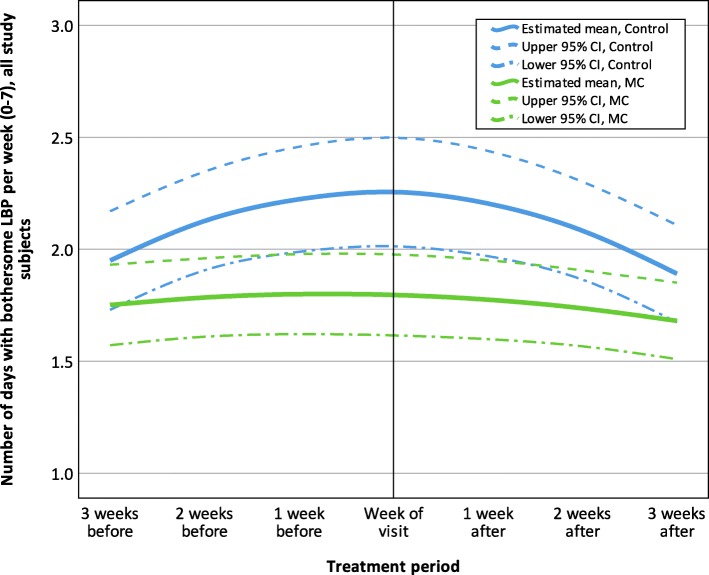
Estimated LBP trajectory 3 weeks before and after visits to a chiropractor, all study participants(*n* = 254), MC, Maintenance Care; LBP, Low Back Pain; CI, Confidence interval; the final GEE model was fitted with an unstructured correlation structure and included the variables: ‘Treatment group’, ‘Time (study period)’, ‘Time (study period)* Time (study period)’, ‘Time (treatment period)’ and ‘Time (treatment period)* Treatment group’

The pain trajectories around the visits were different for the two treatment groups, when all participants were included. The control group showed a steeper increase of pain during the weeks prior to the visit and reached the highest number of days with pain in the week of treatment, when the largest mean difference between groups can be seen (0.46 bothersome (activity-limiting) days with LBP per week; 95% CI =0.16, 0.76). The MC group had a less dramatic trajectory, reporting fewer days with bothersome (activity-limiting) LBP compared to the control group. The pain trajectory for all participants is reported in Fig. 1.

**Fig. 2:**
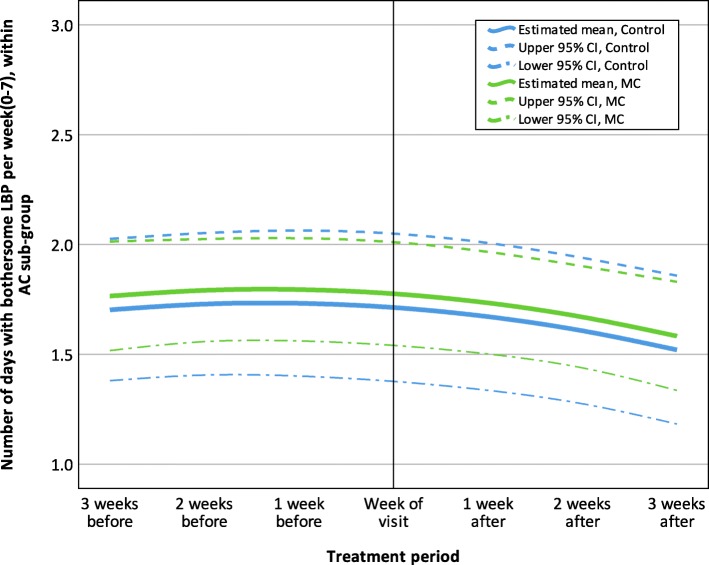
Estimated LBP trajectory 3 weeks before and after visits to a chiropractor, within the Adaptive Coper sub-group (*n* = 80), MC, Maintenance Care; LBP, Low Back Pain; CI, Confidence Interval; AC, Adaptive Coper; The final GEE model was fitted with an unstructured correlation structure and included the variables: ‘Treatment group’, ‘Time (treatment period)’ and ‘Time (treatment period)* Time (treatment period)’

**Fig. 3:**
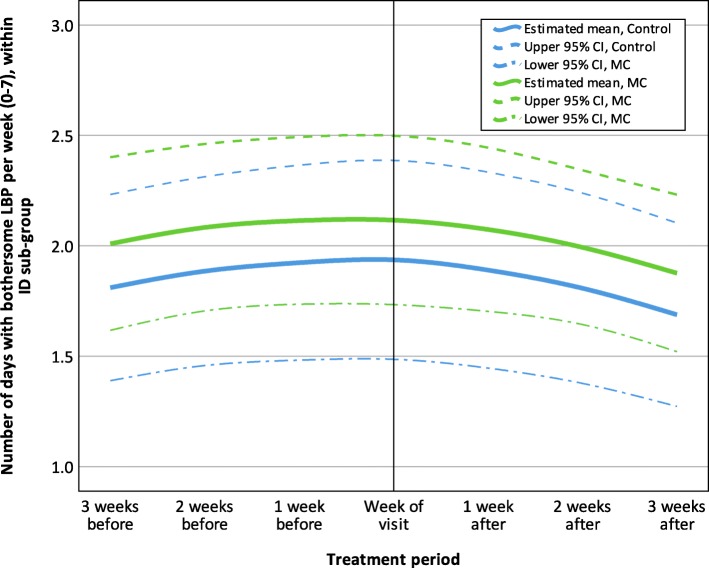
Estimated LBP trajectory 3 weeks before and after visits to a chiropractor, within the Interpersonally Distressed sub-group (*n* = 50), MC, Maintenance Care; LBP, Low Back Pain; CI, Confidence interval; ID, Interpersonally Distressed; The final GEE model was fitted with an unstructured correlation structure and included the variables: ‘Treatment group’, ‘Time (study period)’, ‘Time (study period)*Time (study period)‘and ‘Time (treatment period)’

In the adaptive coper and interpersonally distressed subgroups the estimates across the seven measurements were similar, with no apparent difference between the control and MC groups with regards to their LBP trajectories. The trajectories for adaptive coper and interpersonally distressed subgroups are shown in Figs. 2 and 3.

**Fig. 4:**
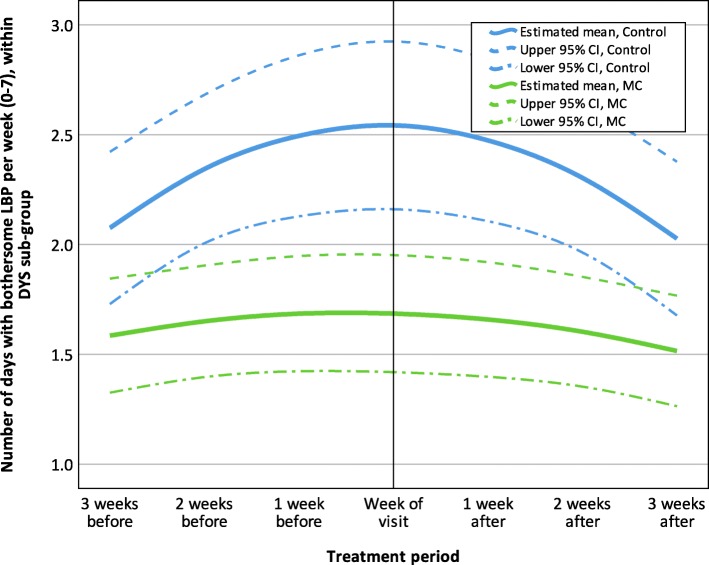
Estimated LBP trajectory 3 weeks before and after visits to a chiropractor, Dysfunctional sub-group (*n* = 73), MC, Maintenance Care; LBP, Low Back Pain; CIDYS, Dysfunctional; the final GEE model was fitted with an unstructured correlation structure and included the variables: ‘Treatment group’, ‘Time (study period)’, ‘Time (treatment period)’ and ‘Treatment group*Time (treatment period)’

The LBP trajectory for the dysfunctional subgroup demonstrates great differences between treatment groups throughout the treatment period, with the largest mean difference around the visit (0.86; 95% CI = 0.39, 1.32). Like the analysis with all participants, the control group had a steep increase in LBP up to the week of the treatment and a decreasing trend after the visit; in comparison the MC group had a flat trajectory. The LBP trajectory for the dysfunctional subgroup is reported in Fig. 4.

In Supplementary Material 1, weekly adjusted mean group differences of days with LBP for the treatment periods for the whole group and all MPI-S subgroups are reported.

### The time to and risk of a new episode following the first recovery period

**Fig. 5:**
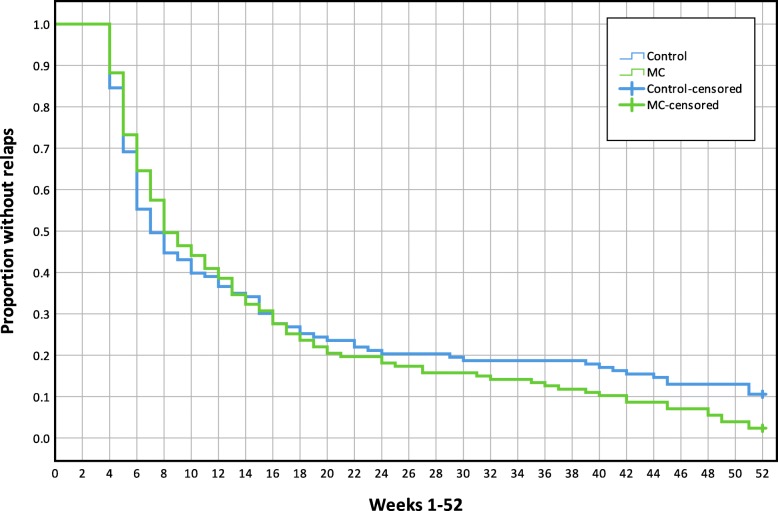
Mean time to new low back pain episodes, Kaplan-Maier plot, all study participants(n = 250). MC, Maintenance Care

**Fig. 6:**
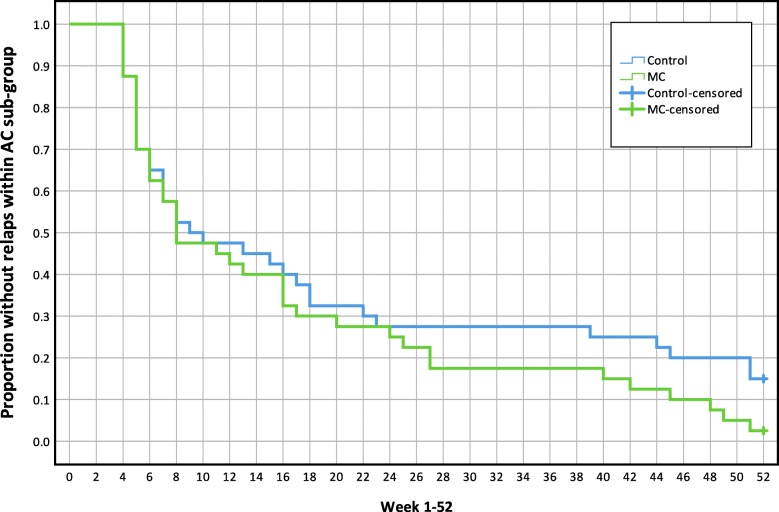
Mean time to new low back pain episodes, Kaplan-Maier plot, Adaptive Coper subgroup (n = 80), MC, Maintenance Care

**Fig. 7:**
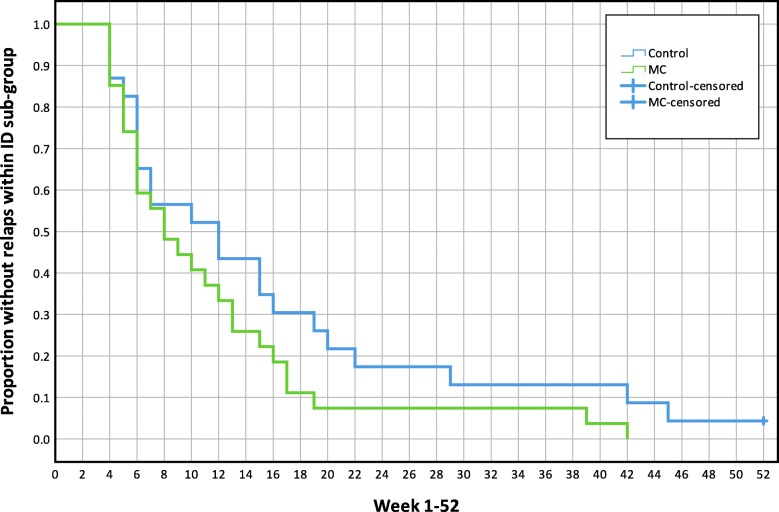
Mean time to new low back pain episodes, Kaplan-Maier plot, Interpersonally Distressed subgroup (*n* = 50). MC, Maintenance Care

**Fig. 8:**
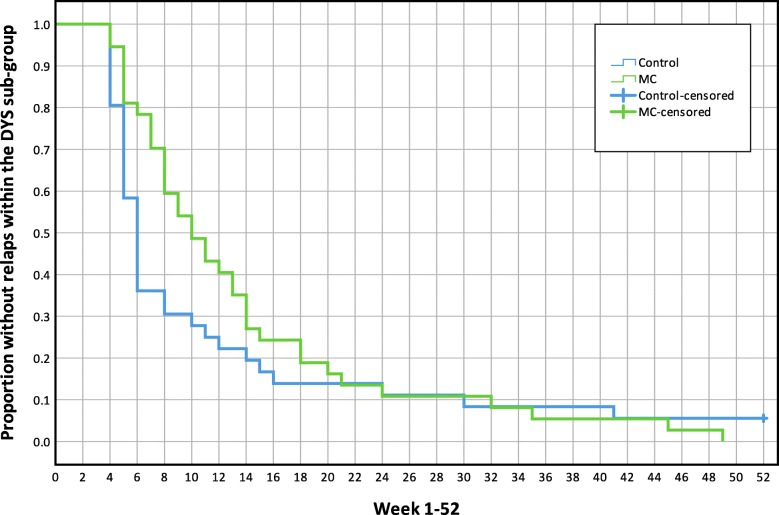
Mean time to new low back pain episodes, Kaplan-Maier plot, Dysfunctional subgroup (*n* = 73). MC, Maintenance Care

There were no mean differences between treatment groups in the Hazard Ratio of new episodes or time-to-event in any of the analyses (all participants or MPI-S subgroups). Results are reported in Table 3. Kaplan-Maier graphs illustrate survival functions for each analysis in Figs. 5, 6, 7 and 8.

**Table 3:** The mean time to event and Hazard Ratio of a new low back pain episode, mean length of consecutive pain-free periods and total number of pain-free weeks

Variable (95% CI)		All participants (*n* = 250)	AC (*n* = 80)	ID (*n* = 50)	DYS (*n* = 73)	
Hazard ratio (MC/Control), HR		**1.1** (0.9, 1.5)	**1.3** (0.8, 2.1)	**1.5** (0.8, 2.7)	**0.8** (0.5, 1.2)	
Time to event, number of weeks to first relapse (single event), mean	MC	15.0 (12.6, 17.4)	16.7 (12.0, 21.5)	11.5 (7.9, 15.1)	13.8 (10.3, 17.2)	
	Control	16.3 (13.4, 19.2)	20.2 (14.4, 26.0	15.8 (10.3, 21.4)	11.4 (7.3, 15.5)	
	Difference	**−1.3** (−5.2, 2.5)	**−3.4** (− 11.1, 4.3)	**−4.3** (− 11.0, 2.4)	**2.4** (− 3.1, 7.9)	
Total number of pain-free weeks during entire study period (between all events), mean.	MC	33.5 (30.1, 36.9)	32.9 (28.4, 37.4)	30.3 (17.9, 42.6)	37.3 (33.0, 41.6)	
	Control	30.9 (28.0, 33.7)	34.8 (29.5, 40.1)	29.2 (23.0, 35.4)	27.4 (22.4, 32.4)	
	Difference	**2.6** (−1.8, 7.0)	**−1.9** (−8.7, 4.9)	**1.0** (− 13.2, 15.3)	**9.8** (3.3, 16.3)	
Length of pain-free periods (weeks between all events), mean	MC	16.4 (14.1, 18.7)	18.3 (13.6, 22.9)	12.4 (8.0, 17.0)	16.1 (12.7, 19.6)	
	Control	17.2 (14.5, 20.0)	21.4 (15.9, 26.9)	15.6 (9.9, 21.3)	12.3 (8.6, 16.1)	
	Difference	**−0.8** (−4.4, 2.8)	**−3.1** (−10.2, 4.0)	**3.1** (− 10.1, 3.9)	**3.8** (− 1.2, 8.8)	

*AC* Adaptive Copers; *ID* Interpersonally Distressed; *DYS* Dysfunctional; *CI* Confidence interval; *HR* Hazard Ratio; *MC* Maintenance Care; An event (*new pain episode)* was defined as a week with ≥2 [2–7] days with bothersome LBP preceded by ≥4 consecutive pain-free weeks

### The length of consecutive pain-free periods and total number of pain-free weeks during the study period

There were no differences between treatment groups in the mean length of pain-free periods or in the mean total number of pain-free weeks when all participants were included in the analysis. When the data were stratified according to the MPI-S subgroups, MC resulted in 9.8 (CI95% 3.3, 16.3) more pain-free weeks (mean) compared to control in the dysfunctional sub-group during the 12-month study period. In the adaptive coper and interpersonally distressed sub-groups the mean differences were small and uncertain (AC -1.9, 95% CI: − 8.7, 4.9; ID 1.0, 95% CI: − 13.2, 15.3). The results are reported in Table 3.

## Discussion

This study is the first to investigate how patients benefit from MC using an in-depth analysis of the clinical course of LBP. It answers questions about how MC affects the number of days with bothersome (activity-limiting) LBP over time by exploring the trajectory around the visit to the chiropractor, the time to and risk of new episodes, and length of pain-free periods (consecutive and total number of).

We found that patients receiving MC had flat LBP trajectories around each new treatment period, as their appointments were unrelated to their symptoms, as opposed to the control group, who were encouraged to call in ‘when needed’. The MC group also had fewer days of bothersome (activity-limiting) LBP than patients receiving the control intervention, who demonstrated more acute episodes.

However, in the adaptive coper and interpersonally distressed groups there were no differences for any of the reported outcomes and the entire difference between MC and control groups was attributed to the dysfunctional subgroup. In the dysfunctional subgroup, there were no differences in the time to/risk of first new episode although MC patients reported more pain-free weeks than the control group. In other words, the clinical course was stabilized by reducing the number of days with LBP per week of each new episode and by increasing the total number of pain-free weeks in the dysfunctional group. As the number of days with bothersome LBP has been shown to correlate with pain intensity [42], it is likely that these patients not only experienced fewer days with LBP but also less intense pain when in remission. These findings are of clinical relevance and may directly affect the delivery of MC in chiropractic practice so that appropriate patients (those with a dysfunctional profile) should be offered MC instead of indiscriminately recommending the procedure to all patients which seems to be the case currently.

The use of weekly SMS with exceptionally few missing data points allows for a detailed analysis of the LBP trajectories with a low risk of recall bias. Therefore, the data used in this trial can be considered robust and of high quality. The inclusion procedure and execution of the MC protocol have been carefully designed to incorporate all the available evidence in the field and to mimic the procedure used in clinical practice as closely as possible. The multi-center pragmatic design with clinics distributed across the country and a high number of involved clinicians allows us to generalize the results to normal clinical practice within the Nordic countries. Although it has been used previously in several trials, measuring the number of days with bothersome (activity-limiting) LBP is novel. A limitation with regards to the clinical relevance of the findings is the lack of knowledge of the exact properties of the primary outcome (number of days with bothersome, activity-limiting, LBP) concerning how it correlates with other health measurements (other than pain intensity). However, the benefit of the measurement is that it reports only the kind of pain that is relevant to the patient. Another potential concern is the way we have classified LBP episodes in this study, which may result in a risk of systematic error that could have underestimated the length and prevalence of LBP episodes. It is known that LBP has a highly individual trajectory and often fluctuates over time rather than being constantly present [43, 44]. We defined our LBP episodes as at least 2 days with pain per week. Presently, no validated definition of a painful event seems to exist; i.e. it is not known for how long pain must be present to be considered a problem by the individual suffering the pain. We defined an event of pain as at least 2 days during a week, assuming that this would represent a problem that was noticeable and relevant.

This study demonstrated that, among dysfunctional patients, MC reduced the number of days of activity limiting LBP in each new episode and people enjoyed more pain-free weeks. At most, the mean difference among dysfunctional patients was 0.86 (CI95%: 0.39, 1.32) fewer days with pain in each new episode while also enjoying 9.8 (3.3, 16.3) more pain free weeks, resulting in a total of 30.0 (95% CI: 36.6, 23.4) fewer days with pain for the entire 52 week period [24]. These estimates are likely to be clinically relevant for the individual and probably cost effective from a societal perspective given that the mean number of visits between MC and control was equal [24]. However, we do not understand the mechanism of this effect. MC may, for example, have a biomechanical effect on joints/tissues by maintaining function and reducing pain sensitivity, but then this should be observed also in the other subgroups of patients. This was not the case and - in fact - one group should *not* receive MC as it worsened their situation [24]. It is therefore more likely that MC has a psychological effect in the group that has worst coping strategies and struggle to manage their pain, whereby meeting and interacting with the clinician reduces the severity of the pain experience. The explanation could also be behavioral, whereby pre-scheduled appointments help people to act more appropriately, i.e. adhere to exercise programs, challenge fearful beliefs and keep active despite pain etc. However, why this would work only for the dysfunctional group is unclear.

Irrespective of the mechanism is seems that the effectiveness of MC among dysfunctional patients could depends on the timing of visits to the chiropractor, i.e. independent of pain ideally before or early in a new episode. If this is an important part of the mechanism, patients with regular LBP episodes could be more successfully scheduled according to a structured MC program because of improved precision in the timing of treatments due to predictability of the pain trajectory. If this rationale holds true these patients may benefit most from the intervention through a reduction of the impact of the most severe periods of pain, perhaps reducing the risk of sick leave, presenteeism and limitations on the activities of daily living.

However, at this point in time the mechanisms behind MC are unknown and remain a question for future projects. Future research should therefore focus on replicating the findings from this trial and understanding why MC has a positive effect on dysfunctional patients but appears to have the opposite effect for adaptive copers. A suitable approach would be to conduct an RCT on dysfunctional patients, randomizing them to a chiropractic maintenance care program or a program consisting of psychological support, advice and reassurance to tease out if it is mainly the physical component of the treatment that ‘works’ or if it is the psychological component that is responsible for this outstanding result. Another important avenue for future research relates to health economic evaluations of MC to better understand and model the societal consequences of MC. To establish if the procedure is cost-effective is imperative for policy makers and health care funders and a key component when implementing the procedure on a large scale.

The message for the patient should be that MC is not a cure that prevents new episodes but rather a management strategy that may reduce bothersome (activity-limiting) LBP over time for a carefully selected group of patients with a dysfunctional profile. Patents who adapt well to their pain, who experience only little interference, have an active lifestyle and do not experience high levels of distress are not suited for MC and should be cared for on an episode to episode basis. Such a message could potentially align expectations to realistic outcomes and may result in a higher degree of trust and a more effective therapeutic alliance between the clinician and the patient, both of which are important for a successful treatment outcome [45–48].

## Conclusion

Chiropractic Maintenance Care reduces the number of days of bothersome (activity-limiting) pain within each new LBP episode among patients classified as dysfunctional (by the MPI-S instrument). MC stabilizes the clinical course and increases the number of pain-free weeks between episodes. Understanding how subgroups of patients are likely to be affected by MC may help align patients’ and clinicians’ expectations with realistic outcomes and can be used as a framework in the selection and execution of appropriate care plans. MC is not a cure that prevents new episodes but rather a management strategy that reduces bothersome (activity-limiting) pain over time for a carefully selected group of patients with recurrent and persistent LBP.

## Supplementary information

**Additional file 1.** Pain trajectories around visits, difference in mean (adjusted) number of days with bothersome pain between groups (Control – MC, 0-7).

## Data Availability

Data cannot be shared publicly for legal reasons. Data will be available upon request after permission is granted from the Karolinska Institutet’s Ethics Review Board in Stockholm: kansli@stockholm.epn.se. Inquiries for data access should first be sent to irene.jensen@ki.se, who will then contact the ethics board for permission to openly share the data.

## Abbreviations

LBP: Low Back Pain
MC: Chiropractic maintenance care
MPI-S: Swedish version of West Haven-Yale Multidimensional Pain Inventory
