# Supplementary material for: The Nordic maintenance care program: maintenance care reduces the number of days with pain in acute episodes and increases the length of pain free periods for dysfunctional patients with recurrent and persistent low back pain - a secondary analysis of a pragmatic randomized controlled trial
Source: Chiropr Man Therap. 2020 Apr 21;28:19. doi: 10.1186/s12998-020-00309-6 (PMC7171853; doi:10.1186/s12998-020-00309-6)
Supplement: Supplementary file 1 — Additional file 1. Pain trajectories around visits, difference in mean (adjusted) number of days with bothersome pain between groups (Control – MC, 0-7). [file 12998_2020_309_MOESM1_ESM.pdf]

Supplementary material 1: Pain trajectories around visits, difference in mean (adjusted) number of days with bothersome pain between groups (Control – MC, 0-7).

	All subjects				Adaptive Coper				Interpersonally Distressed				Dysfunctional			
**Treatment period**	**Diff.**	**L95CI**	**U95CI**	**p**	**Diff.**	**L95CI**	**U95CI**	**p**	**Diff.**	**L95CI**	**U95CI**	**p**	**Diff.**	**L95CI**	**U95CI**	**p**
3 weeks before	0.20	-0.09	0.48	0.171	-0.06	-0.47	0.35	0.763	-0.20	-0.77	0.38	0.499	0.49	0.06	0.92	0.026
2 weeks before	0.34	0.06	0.62	0.017	-0.06	-0.46	0.34	0.759	-0.20	-0.77	0.37	0.497	0.69	0.27	1.12	0.001
1 week before	0.42	0.13	0.72	0.005	-0.06	-0.47	0.34	0.763	-0.19	-0.77	0.39	0.521	0.81	0.36	1.26	<0.001
**Week of visit**	**0.46**	**0.16**	**0.76**	**0.003**	**-0.06**	**-0.47**	**0.35**	**0.765**	**-0.18**	**-0.77**	**0.41**	**0.551**	**0.86**	**0.39**	**1.32**	**<0.001**
1 week after	0.43	0.14	0.72	0.004	-0.06	-0.47	0.35	0.764	-0.18	-0.76	0.39	0.533	0.82	0.37	1.27	<0.001
2 weeks after	0.35	0.07	0.62	0.014	-0.06	-0.47	0.34	0.762	-0.19	-0.74	0.37	0.513	0.70	0.28	1.12	0.001
3 weeks after	0.21	-0.07	0.49	0.135	-0.06	-0.48	0.36	0.770	-0.19	-0.74	0.36	0.500	0.51	0.08	0.94	0.020

MC, Maintenance Care; **Diff.**, Difference between groups; **L95CI**, Lower 95% confidence interval; **U95CI**, upper 95% confidence interval; **p**, p-value.
